# The Selectivity of Fosfosal for STAT5b over STAT5a is Mediated by Arg566 in the Linker Domain

**DOI:** 10.1002/cbic.202000111

**Published:** 2020-05-08

**Authors:** Julian Gräb, Thorsten Berg

**Affiliations:** ^1^ Leipzig University, Institute of Organic Chemistry Johannisallee 29 04103 Leipzig Germany

**Keywords:** inhibitors, protein-protein interactions, SH2 domain, transcription factors

## Abstract

Fosfosal is the O‐phosphorylated derivative of salicylic acid, with documented clinical use as a prodrug for the treatment of inflammatory diseases. We recently discovered that fosfosal itself inhibits the protein‐protein interaction domain, the SH2 domain, of the tumor‐related transcription factor STAT5b. Here, we demonstrate that fosfosal is selective for STAT5b over its close homologue STAT5a. This selectivity is mediated by the STAT5b residue Arg566, located in the SH2 domain‐adjacent linker domain. Our data provide further evidence for the role of the STAT linker domain in determining the activity of small molecules against the SH2 domain. We present a refined binding model for fosfosal and STAT5b, which can serve as the basis for the development of fosfosal‐based STAT5b inhibitors.

## Graphical abstract

**On the edge**: Fosfosal, a prodrug for the treatment of inflammatory diseases, inhibits the tumor‐related transcription factor STAT5b with selectivity over the close homologue STAT5a. Comparative analysis between wild‐type and mutant STAT5 proteins revealed that fosfosal binds not only to the protein‐protein interaction domain, the SH2 domain (green), but also to Arg566 in the adjacent linker domain (magenta).

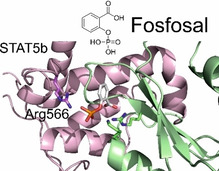

STATs (signal transducers and activators of transcription) are dimeric transcription factors which convey signals from the cell membrane to the nucleus.^1^ Two forms of the family member STAT5 exist, STAT5a and STAT5b, which exhibit 94 % sequence identity on the amino acid level.^2^ Despite this high degree of similarity, STAT5a and STAT5b exhibit some nonredundant functions.^3^ Although both STAT5 proteins are frequently constitutively activated in human cancers,^4^ STAT5b has been shown to be the main driver of tumor cell proliferation in both squamous cell carcinoma^5^ and Bcr‐Abl‐positive leukemia cells.^6^ In NPM1‐ALK–expressing T‐cell lymphomas, STAT5b signaling promotes tumor growth, but STAT5a acts as a tumor suppressor.^7^ In contrast, inhibition of STAT5a, but not STAT5b, increases the differentiation of osteoblasts in human bone marrow‐derived stromal cells, suggesting selective inhibition of STAT5a as a therapeutic modality against age‐related osteoporosis.^8^

The role of STAT5 in cancer has encouraged the development of a number of STAT5 inhibitors.^9^ These are thought to act on the STAT5 protein‐protein interaction domain, the Src homology 2 (SH2) domain. However, despite the known non‐identical roles of STAT5a and STAT5b, the differential effects of most STAT5 inhibitors on the two proteins have not been tested. Our group recently developed the catechol bisphosphates Stafib‐1,^10^ Stafib‐2,^11^ and Capstafin^12^ as the first small molecules that inhibit STAT5b with selectivity over STAT5a,^13^ and the *m*‐terphenyl phosphate Stafia‐1 as the first small molecule that inhibits STAT5a with selectivity over STAT5b.^14^

The starting point for the development of our catechol bisphosphate‐based STAT5b inhibitors^10^ was fosfosal (O‐phosphorylated salicylic acid, Figure 1A), which we identified as a STAT5b SH2 domain inhibitor during high‐throughput screening of natural products and known bioactive compounds.9b Fosfosal (Disdolen®, Uriach, Spain) has been in clinical use as a phosphate prodrug of salicylic acid for treatment of inflammatory diseases.^15^ The selectivity of fosfosal itself for STAT5b over STAT5a was not investigated in the original study,9b because the competitive binding assay against STAT5a^10^ had not yet been developed.

**Figure 1:**
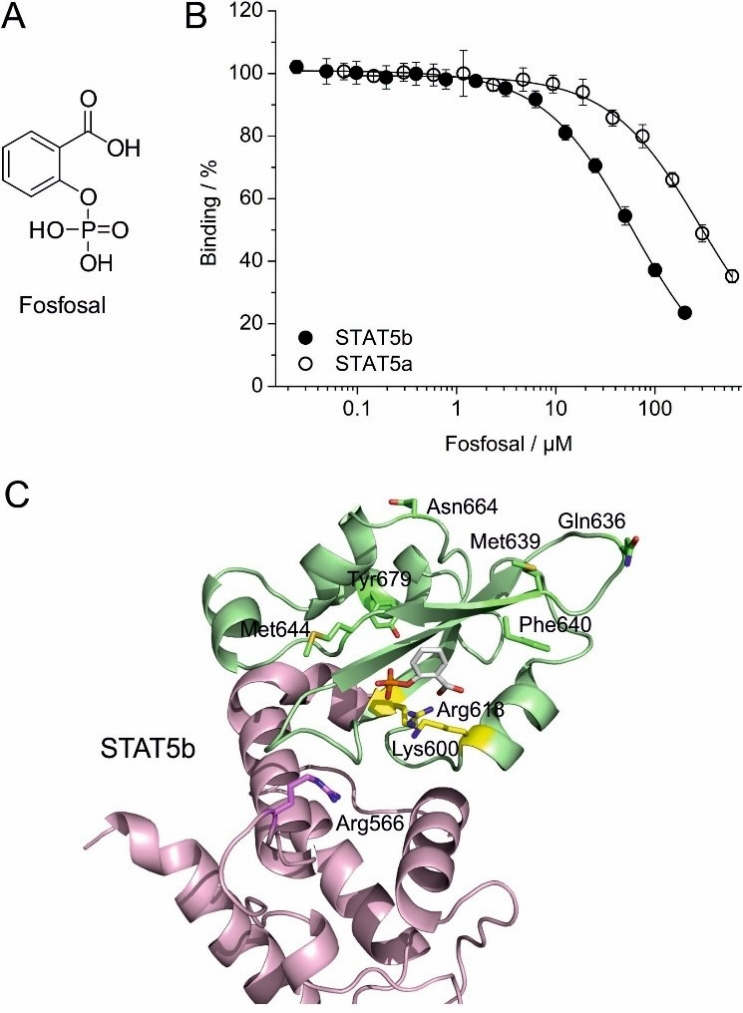
A) Structure of fosfosal. B) Activity of fosfosal against wild‐type STAT5b and STAT5a in competitive FP assays. C) Previously published docking pose of fosfosal into STAT5b generated using AutoDock Vina.9b Linker domain: magenta; SH2 domain: green. The side chains of the conserved Lys600 and Arg618 are shown with carbon atoms in yellow; the carbon atoms of the side chains of the divergent amino acids in the SH2 domain and in position 566 of the linker domain are colored according to the corresponding domain.

Here, we provide a detailed activity analysis of fosfosal against STAT5a and STAT5b wild‐type and point mutant proteins. Our data lead to a refined binding model for fosfosal and STAT5b, which will support the rational development of fosfosal‐based STAT5b inhibitors, and highlights the relevance of the STAT linker domain for the function of the adjacent SH2 domain.

Analysis of fosfosal in a fluorescence polarization (FP)‐based competitive binding assay indicated a fivefold weaker activity against STAT5a (*K*_i_=148±9 μM) than STAT5b (*K*_i_=29.3±1.8 μM, Figure 1B, Table 1, and Table S1 in the Supporting Information). This result cannot be explained by the previously published docking model of fosfosal and STAT5b (Figure 1C),9b which postulated interactions between the negatively charged groups of fosfosal and the STAT5b amino acids Lys600 and Arg618 (Figure 1C) to be the main determinants of protein binding. Because Lys600 and Arg618 are conserved between STAT5b and STAT5a, they cannot govern binding specificity. The published binding pose was generated by docking fosfosal into a rigid STAT5b homology model based on the crystal structure of STAT5a^16^ using AutoDock Vina,^17^ which does not account for protein flexibility.

**Table 1:** Activities of fosfosal against wild‐type and mutant STAT5 proteins.^[a]^

Protein	*K*_i_ [μM] or % inhibition at 600 μM
STAT5b	29.3±1.8 μM
STAT5a	148±9 μM
STAT5b Arg566Trp	24±2 % inhibition
STAT5a Trp566Arg	10.2±0.7 μM
STAT5b‐6M	10.7±0.6 μM
STAT5b‐7M	189±3 μM
STAT5b Arg566Ala	22±3 % inhibition
STAT5b Arg566Glu	no inhibition
STAT5b Met644Lys	17.2±0.9 μM
STAT5a Trp566Arg/Lys644Met	12.3±0.6 μM

^[a]^ Inhibition constants *K*_i_ have been calculated from the IC_50_ values shown in Supporting Table S1, using the published equation.^19^

We recently identified Arg566 in the STAT5b linker domain as the main determinant of the STAT5b selectivity of catechol bisphosphates.^18^ Although Arg566 appears to be too distant to interact with fosfosal as bound to the SH2 domain of our original homology model (Figure 1C), Arg566 is located in a flexible loop which can shift towards the SH2 domain.^18^ To investigate whether Arg566 is also responsible for the moderate selectivity of fosfosal for STAT5b over STAT5a, we tested the activity of fosfosal against the point mutant protein STAT5b Arg566Trp in a fluorescence polarization assay. Activity against STAT5b Arg566Trp, in which the arginine at position 566 of STAT5b was swapped for the tryptophan at position 566 of STAT5a (Figure S1), was drastically decreased (24±2 % inhibition at 600 μM, the highest concentration tested) compared to wild‐type STAT5b (*K*_i_=29.3±1.8 μM, Figure 2A). Conversely, the activity of fosfosal against the reverse cross‐over mutant STAT5a Trp566Arg was increased by 14‐fold (*K*_i_=10.2±0.7 μM) compared to wild type STAT5a (*K*_i_=148±9 μM, Figure 2A, Tables 1 and S1). These data indicate that the amino acid at position 566 of the linker domain is crucial for inhibition by fosfosal. We also tested fosfosal against the STAT5b mutant Gln636Pro/Met639Asn/Phe640Leu/Met644Lys/Asn664Ser/Tyr679Phe (dubbed STAT5b‐6M), in which all six of the amino acids which differ from the SH2 domain of STAT5a were mutated to the corresponding STAT5a residues (Figure S1).^18^ The activity of fosfosal against STAT5b‐6M (*K*_i_=10.7±0.6) was not reduced to the level of wild type STAT5a (*K*_i_=148±9 μM), but instead increased almost 3‐fold compared to wild type STAT5b (*K*_i_=29.3±1.8 μM, Figure 2A, Tables 1 and S1). Additionally, we tested fosfosal against the STAT5b mutant Arg566Trp/Gln636Pro/Met639Asn/Phe640Leu/Met644Lys/Asn664Ser/Tyr679Phe (dubbed STAT5b‐7M),^18^ in which the Arg566Trp mutation was introduced in STAT5b‐6M. STAT5b‐7M showed substantially decreased affinity for fosfosal (*K*_i_=189±3 μM) compared to STAT5b‐6M (*K*_i_=10.7±0.6 μM, Figure 2A, Tables 1 and S1). Taken together, these results indicate that the preference of fosfosal for STAT5b is mediated by the amino acid in position 566 of the STAT5a/b linker domain, and not by the divergent amino acids in the SH2 domain.

**Figure 2:**
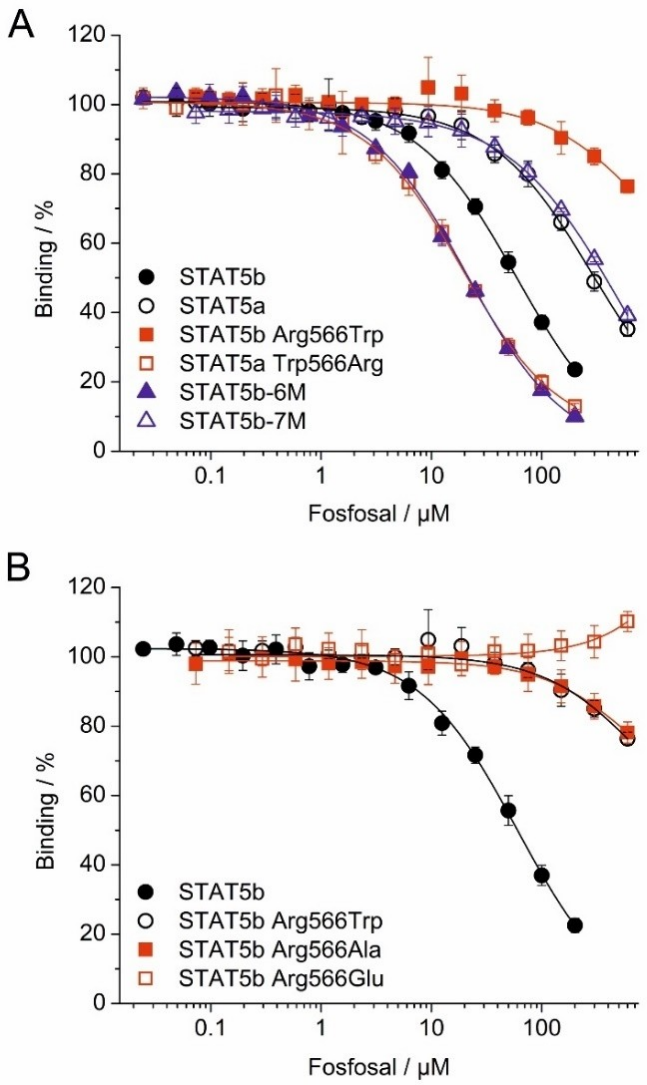
A) Activity of fosfosal in FP assays against wild‐type STAT5b, wild‐type STAT5a, STAT5b Arg566Trp, STAT5a Trp566Arg, STAT5b‐6M, and STAT5b‐7M. B) Activity of fosfosal in FP assays against wild‐type STAT5b, STAT5b Arg566Trp, STAT5b Arg566Ala, and STAT5b Arg566Glu.

The strongly reduced activity of fosfosal against STAT5b Arg566Trp (24±2 % inhibition at 600 μM) as compared to wild‐type STAT5b (*K*_i_=29.3±1.8 μM, Figure 2A) may result from the absence of an electrostatic interaction between one of the negatively charged groups of fosfosal and the guanidinium group of the side chain of arginine, or be caused by the presence of the bulky indole side chain of tryptophan. To differentiate between these two possibilities, we tested the mutant proteins STAT5b Arg566Ala and STAT5b Arg566Glu.^18^ While the activity of fosfosal against STAT5b Arg566Ala (22±3 % inhibition at 600 μM) was similarly low to its activity against STAT5b Arg566Trp (24±2 % inhibition at 600 μM), there was no inhibition of STAT5b Arg566Glu (Figure 2B, Tables 1 and S1). These data strongly suggest that the reduced binding of fosfosal to STAT5b Arg566Trp is not caused by the presence of the indole side chain of Trp566, but by the absence of the positively charged side chain of STAT5b Arg566.

To rationalize our experimental data, we docked fosfosal into a previously‐described refined STAT5b homology model, in which the flexible loop in the STAT5b linker domain had been shifted towards the SH2 domain.^18^ We allowed side chain flexibility for amino acids in the vicinity of the putative binding site, using the newly developed docking program AutoDock FR.^20^ This identified a docking pose for fosfosal which was consistent with the experimental data (Figure 3). In this docking pose, the negatively charged groups of fosfosal interact not only with Lys600 and Arg618 in the SH2 domain, but also with Arg566 in the linker domain of STAT5b. The conserved SH2 domain amino acids Ser620 and Ser622, which form hydrogen bonds with the phosphate group of phosphotyrosine‐containing peptide sequences in crystal structures of STAT proteins,^21^ are expected to be engaged in hydrogen bond networks with the carboxylate and the phosphate group of fosfosal.

**Figure 3:**
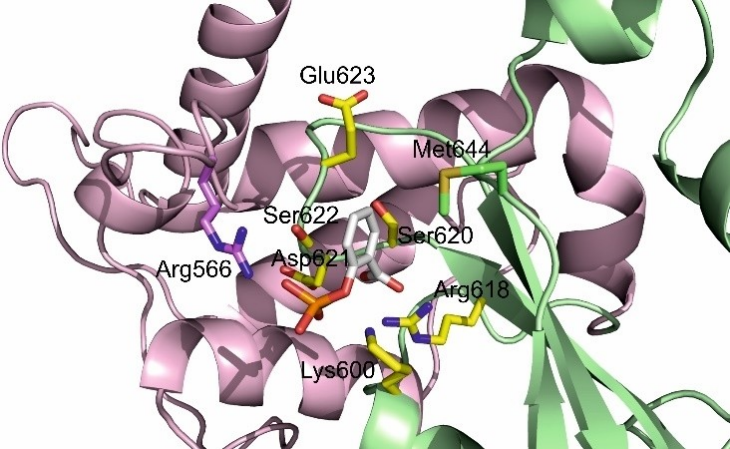
Docking of fosfosal into a homology model of STAT5b, in which the linker domain loop bearing Arg566 is shifted towards the SH2 domain, using AutoDock FR. The colored amino acid side chains were defined as flexible in the docking process. Linker domain: magenta; SH2 domain: green. The side chains of the conserved amino acids are shown with carbon atoms in yellow; the side chains of the divergent amino acids Arg566 and Met644 are shown with carbon atoms colored like the corresponding domain. The figure was generated using PyMOL.^22^

An interesting observation is that the activity of fosfosal against the mutants which comprise the STAT5a SH2 domain together with an arginine in position 566, STAT5a Trp566Arg (*K*_i_=10.2±0.7 μM) and STAT5b‐6M (*K*_i_=10.7±0.6 μM), is higher than the activity against wild‐type STAT5b (*K*_i_=29.3±1.8 μM, Figure 2A, Tables 1 and S1). Similar observations have been made for catechol bisphosphates^18^ and the nucleoside triphosphates ATP and GTP,^23^ which are also more active against these two mutants than against wild‐type STAT5b. From the new docking model of fosfosal (Figure 3), it appeared that the side chain of the divergent STAT5a/b amino acid in position 644 is in proximity to the small‐molecule binding site. Although the Met644 of STAT5b might provide a contribution to binding of fosfosal, the Lys644 present in STAT5a seems more likely to do so, either through strong cation‐π interactions with fosfosal's benzene ring, or through electrostatic interactions with the negatively charged groups of fosfosal. In order to investigate the role of the divergent amino acids in position 644, we created the point mutant protein STAT5b Met644Lys, and found that this was indeed inhibited by fosfosal to a higher extent (*K*_i_=17.2±0.9 μM) than wild‐type STAT5b (*K*_i_=29.3±1.8 μM, Figure 4, Tables 1 and S1). Conversely, the double mutant STAT5a Trp566Arg/Lys644Met was inhibited to a slightly lesser extent (*K*_i_=12.3±0.6 μM) than the single mutant STAT5a Trp566Arg (*K*_i_=10.2±0.7 μM, Figure 4, Tables 1 and S1). This indicates that the amino acid in position 644 does provide a contribution to fosfosal binding. However, the residual activity difference between both STAT5a Trp566Arg (*K*_i_=10.2±0.7 μM) and STAT5b‐6M (*K*_i_=10.7±0.6 μM), and STAT5b Met644Lys (*K*_i_=17.2±0.9 μM) suggests that one or more of the remaining five divergent amino acids in the STAT5a SH2 domain also contributes to the higher activity of fosfosal against STAT5a Trp566Arg and STAT5b‐6M.

**Figure 4:**
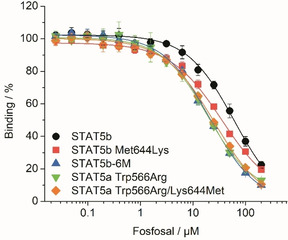
Activity of fosfosal in FP assays against wild‐type STAT5b, STAT5b Met644Lys, STAT5b‐6M, STAT5a Trp566Arg, and STAT5a Trp566Arg/Lys644Met.

In summary, we show that the STAT5b inhibitor fosfosal exhibits fivefold selectivity over the highly homologous STAT5a, and that this selectivity is mediated by Arg566 in the STAT5b linker domain. The refined binding mode of fosfosal presented here will facilitate the development of improved fosfosal‐based STAT5b inhibitors, and is consistent with the binding mode of catechol bisphosphate‐based STAT5b inhibitors.^18^ In both cases, an additional negatively charged group in the *ortho*‐position of the phenyl phosphate moiety (a carboxylate in case of fosfosal, or a second phosphate group in case of catechol bisphosphates) mediates STAT5b selectivity by allowing concomitant targeting of both the SH2 domain and Arg566. Our study reveals that the STAT5b‐selectivity of catechol bisphosphate‐based inhibitors has its origins in the lead structure fosfosal, and provides further evidence for a contribution of the STAT linker domain to the function of the SH2 domain.^24^

## Conflict of interest

The authors declare no conflict of interest.

## Supporting information

As a service to our authors and readers, this journal provides supporting information supplied by the authors. Such materials are peer reviewed and may be re‐organized for online delivery, but are not copy‐edited or typeset. Technical support issues arising from supporting information (other than missing files) should be addressed to the authors.

Supplementary
