# Supplementary material for: The Selectivity of Fosfosal for STAT5b over STAT5a is Mediated by Arg566 in the Linker Domain
Source: Chembiochem. 2020 May 8;21(16):2264–7. doi: 10.1002/cbic.202000111 (PMC7496286; doi:10.1002/cbic.202000111)
Supplement: Supplementary file 1 — Supplementary [file CBIC-21-2264-s001.pdf]

ChemBioChem

Supporting Information

The Selectivity of Fosfosal for STAT5b over STAT5a is Mediated by Arg566 in the Linker Domain

Julian Gräb and Thorsten Berg*

Figure S1

SH2 domains (amino acids 593-683)

STAT5a: AILGFVNKQQAHDLLINKPDGTFLLRFSDSEIGGITIAWKFDS**P**ER**NL**WNL**K**PFTTRDFSIRSLADRLGDL**S**YLIYVFPDRPKDEV**F**SKYY

STAT5b: AILGFVNKQQAHDLLINKPDGTFLLRFSDSEIGGITIAWKFDS**Q**ER**MF**WNL**M**PFTTRDFSIRSLADRLGDL**N**YLIYVFPDRPKDEV**Y**SKYY

Linker domains (amino acids 471-592)

STAT5a:

HGSQD**H**NATATVLWDNAFAEPGRVPFAVPDKVLWPQLCEALNMKFKAEVQSNRGLTKENLVFLAQKLFNNSSSHLEDYSGLSVSWSQFNRENLPG**W**NYTF
WQWFDGVMEVLKKH**H**KPHWNDG

STAT5b:

HGSQD**N**NATATVLWDNAFAEPGRVPFAVPDKVLWPQLCEALNMKFKAEVQSNRGLTKENLVFLAQKLFNNSSSHLEDYSGLSVSWSQFNRENLPG**R**NYTF
WQWFDGVMEVLKKH**L**KPHWNDG

Figure S1. Amino acid sequence of the SH2 domains (upper panel) and linker domains (lower panel) of STAT5a and STAT5b.[1]

Table S1: Activities of fosfosal against wild-type and mutant STAT5 proteins. N/A: not applicable.

Protein	IC_{50} (µM)
STAT5b	59.5 ± 3.6 µM
STAT5a	299 ± 17 µM
STAT5b Arg566Trp	N/A
STAT5a Trp566Arg	21.0 ± 1.5 µM
STAT5b-6M	21.3 ± 1.2 µM
STAT5b-7M	379 ± 7 µM
STAT5b Arg566Ala	N/A
STAT5b Arg566Glu	N/A
STAT5b Met644Lys	36.6 ± 2.0 µM
STAT5a Trp566Arg/Lys644Met	24.5 ± 1.4 µM

Reagents

Cloning and expression of wild-type STAT5a/b proteins and the mutants STAT5b Arg566Trp, STAT5b Arg566Ala, STAT5b Arg566Glu, STAT5a Trp566Arg, STAT5b-6M, and STAT5b-7M have been described previously.[1-2] Similarly, the protein point mutants STAT5b Met644Lys and STAT5a Trp566Arg/Lys644Met were generated by following the QuikChange site-directed mutagenesis protocol (Agilent Genomics). Proteins were purified over His-bind resin twice as previously described.[1] Synthesis of fosfosal has been described.[3]

Fluorescence polarization assays

The ability of test compounds to displace 5-carboxyfluorescein-GY(PO_3H_2)LVLDKW (final concentration: 10 nM) from the SH2 domain of STAT5a/b proteins was analyzed. Proteins were used at the following final concentrations: STAT5b (K_d = 82 or 95 nM): 82 or 95 nM, STAT5a (K_d = 147 nM): 147 nM, STAT5a Trp566Arg (K_d = 43 nM): 43 nM, STAT5b Arg566Trp (K_d = 289 nM): 289 nM, STAT5b-6M (K_d = 85 nM): 85 nM, STAT5b-7M (K_d = 246 nM): 246 nM, STAT5b Arg566Ala (K_d = 567 nM): 567 nM, STAT5b Arg566Glu (K_d = 2 µM): 2 µM, STAT5a Trp566Arg/Lys644Met (K_d = 80 nM): 80 nM, STAT5b Met644Lys (K_d = 26 nM): 26 nM. Fosfosal was applied in dilution series comprising the following concentrations: 0.024 – 200 µM (STAT5b, STAT5a Trp566Arg, STAT5a Trp566Arg/Lys644Met, STAT5b-6M, STAT5b Met644Lys), 0.073 – 600 µM (STAT5a, STAT5b Arg566Ala, STAT5b Arg566Trp, STAT5b-7M) and 0.146 – 600 µM (STAT5b Arg566Glu), respectively. The assays were performed in buffer containing 10 mM Tris, 50 mM NaCl, 1 mM DTT, 1 mM EDTA and 0.1% (v/v) NP-40. Fosfosal was dissolved in DMSO. STAT5a/b proteins and fosfosal were incubated at room temperature for 1 h before adding peptide. Samples were transferred to 384-well microtiter plates and fluorescence polarization was read after a further 1 h incubation time. All measurements were performed in triplicate (n=3), except for STAT5b (n=6) and STAT5a Trp566Arg (n=4). Percent binding was calculated based on a logarithmic curve fit using OriginPro 8G software. IC_{50} values were converted to K_i values using the published equation.[4]

Supporting references

[1] J. Gräb, A. Berg, L. Blechschmidt, B. Klüver, S. Rubner, D. Y. Fu, J. Meiler, M. Gräber, T. Berg, *ACS Chem. Biol.* **2019**, *14*, 796-805.

[2] N. Elumalai, A. Berg, K. Natarajan, A. Scharow, T. Berg, *Angew. Chem. Int. Ed.* **2015**, *54*, 4758-4763.

[3] M. Gräber, W. Janczyk, B. Sperl, N. Elumalai, C. Kozany, F. Hausch, T. A. Holak, T. Berg, *ACS Chem. Biol.* **2011**, *6*, 1008-1014.

[4] Z. Nikolovska-Coleska, R. Wang, X. Fang, H. Pan, Y. Tomita, P. Li, P. P. Roller, K. Krajewski, N. G. Saito, J. A. Stuckey, S. Wang, *Anal. Biochem.* **2004**, *332*, 261-273.
